# Secondary Endovascular Conversions for Failed Open Repair

**DOI:** 10.1055/s-0043-1774724

**Published:** 2023-11-10

**Authors:** Ryan Gouveia e Melo, Paolo Spath, Jan Stana, Carlota F. Prendes, Konstantinous Stavroulakis, Barbara Rantner, Maximilian Pichlmaier, Nikolaos Tsilimparis

**Affiliations:** 1 Vascular Surgery Department, Ludwig Maximilian University Hospital, Munich, Germany; 2 Cardiac Surgery Department, Ludwig Maximilian University Hospital, Munich, Germany

**Keywords:** late complication, open aortic repair, endovascular aortic repair, endovascular salvage

## Abstract

Late aortic and graft-related complications after open aortic repair are not infrequent and a significant number of them are missed, diagnosed at a very late stage, or present as urgent complications such as aortic rupture or aorto-enteric fistula. Once a late complication is diagnosed and reintervention is necessary, both open and endovascular strategies are possible. Open reintervention is complex and usually associated with very high rates of morbidity and mortality. Endovascular techniques may offer several solutions for these cases, which may be tailored to the patient and specific complication. In this review, we aim to summarize current indications, options, and strategies for endovascular salvage after failed or complicated open surgical repair.

## Introduction

Open surgical aortic repair usually involves complete or partial resection of the diseased aortic segment followed by replacement with a synthetic or homograft. 1 2 Contrary to endovascular aortic repair, in which the disease segment is excluded from the circulation but not resected, requiring lifetime disease progression surveillance, follow-up after open repair is not as standardized and is frequently overlooked. 3 4 5 This leads to a significant number of late complications after open repair being missed, diagnosed at a very late stage, or even presenting as urgent complications such as aortic rupture or aorto-enteric fistula. To address this, some societies have advocated for long-term follow-up with computed tomography (CT) imaging at 5 years after open aortic repair. 1 2

Moreover, late complications following open surgical repair have been reported to occur in non-negligible rates. Para-anastomotic aneurysms have been reported in up to 12% of patients following open abdominal aortic aneurysm (AAA) repair. 1 Disease progression after previously treated aortic aneurysms is also expected to occur in approximately 5% of patients after 10 years. 6

Once a late complication is diagnosed and reintervention is necessary, both open and endovascular strategies are possible solutions. Open reintervention is complex, since the surgical dissection of scarred tissues involves higher risk of visceral organ injury, uncontrolled bleeding, collateral target artery damage, and higher rates of postoperative infections. 1 An alternative option in these cases, especially if complications occur at the aortic level (contrary to lower limb and femoral artery–related complications, for example) is to convert to endovascular repair. Endovascular repair may be performed as bridging strategy in an emergency case or as a definitive repair. 1

In this review we summarize current indications, options, and strategies for endovascular salvage after failed or complicated open surgical repair.

## Endovascular Repair of Late Abdominal Aortic Open Repair Complications

### Para-anastomotic Aneurysms after Open Abdominal Aortic Aneurysm Repair

The true rate of proximal aortic para-anastomotic aneurysms following open AAA repair is unknown since a significant number of these patients are irregularly followed. A recent study by Serizawa et al 5 found, in a cohort of patients with mean follow-up of 7.1 years, under routine follow-up, an incidence of para-anastomotic aneurysms of 2.2 and 3.6% at 5 and 10 years, respectively. However, rates as high as 10 to 15% have been described. 6 7

Several studies have been published demonstrating the use of endovascular repair in these situations. 7 8 Spanos et al 7 have published a systematic review and meta-analysis analyzing the outcomes of endovascular treatment of para-anastomotic aneurysms after abdominal aortic surgery. Overall, 18 studies were included, totaling 433 patients. Mean time from index surgery to diagnosis was 10 years. Endovascular techniques varied from proximal cuffs, tube grafts, iliac extensions, parallel graft techniques, fenestrated and branched repairs, or combinations of techniques. The most common techniques were standard bifurcated grafts (23.7%) followed by fenestrated endografts (23.4%). Technical success was 97.8% with 1.4% mortality rate and low rate of complications.

One technical challenging aspect following proximal para-anastomotic aneurysms tends to be the lack of proximal sealing, since most grafts are sutured to the juxtarenal aorta, making it necessary to involve the pararenal/visceral aorta for a durable endovascular repair. Tshomba et al 9 showed that, in proximal para-anastomotic aneurysms following open AAA repair, less than 20% of repairs were feasible with a simple endovascular aneurysm repair device, and even if feasible, a significant number of reinterventions were necessary during follow-up. Additionally, depending on the length of the main body of the graft, one may have challenges in fitting a fenestrated cuff in a short segment between the graft bifurcation and the visceral vessels. To address this, an important technical note might be, when performing open repair of the abdominal aorta, to leave enough main body fabric length for possible future repairs.

### Disease Progressions after Abdominal Aortic Aneurysm Repair

Proximal disease extension, synchronous or metachronous aneurysms may also lead for further necessary operations following initial AAA repair. 6 10 Plate et al 6 showed that in a cohort of 1,112 patients at 5 years after repair, 5% presented with aneurysm progression. In these cases, proximal extension with a fenestrated or branched device has been shown to produce acceptable results. In a multicenter study analyzing 108 cases, technical success was 93%, spinal cord ischemia occurred in 6.5%, (3.7% permanent), 30-day mortality was 4%, and there was no late aneurysm-related mortality, with freedom from reintervention at 5 years of 74%. 11 In patients who have undergone an open abdominal repair who progress to a proximal thoracoabdominal aortic aneurysm (TAAA), since, historically, staging of operation has been shown to lower the risk of spinal cord ischemia, need for spinal fluid drainage in these patients is questionable. 12

A recent meta-analysis has also shown that, in patients with a known AAA, 19.2% will have a synchronous or metachronous thoracic aortic aneurysm, with even higher rates (30.7%) in women. 10 Endovascular repair in these patients may simplify the procedure, if a simple thoracic endovascular aneurysm repair (TEVAR) can be offered, or by avoiding open reintervention in the hostile abdomen if a thoracoabdominal repair is needed.

**Fig. 1:**
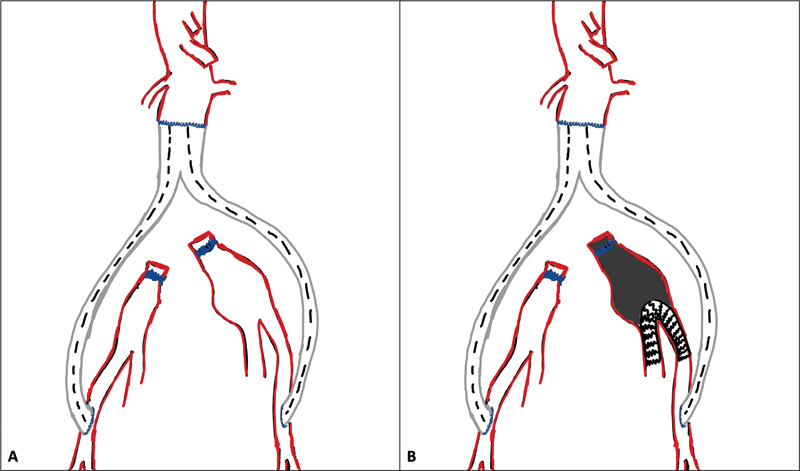
Schematic representation of the use of the “banana technique” to exclude a residual common iliac aneurysm after open abdominal aortic repair with an aorto-bi-femoral configuration. In this case, to preserve the hypogastric artery, a covered self-expanding stent graft is placed from the external to the internal iliac artery, thus excluding the common iliac and resembling a banana in shape. ( **A** ) Schematic representation of a residual common iliac aneurysm after open aortic repair with aorto-bifemoral reconstruction and common iliac artery proximal ligation with late degeneration. ( **B** ) Schematic representation of the “banana technique” excluding the common iliac artery aneurysm with a self-expanding covered stent from the external to the internal iliac artery.

One less common but also possible progression is aneurysmal degeneration of the common iliacs in cases where the repair was performed in an aorto-bi-femoral configuration with distal aortic or proximal iliac ligation. In these cases, to preserve the hypogastric arteries, one may perform the so called “banana technique” with placement of a covered self-expanding stent graft from the external to the internal iliac artery, thus excluding the common iliac ( Fig. 1 ). 13 14

### Aortoenteric Fistula with Urgent Bleeding

One of the most feared and deadly complications after abdominal aortic open repair is graft infection with an aorto-enteric fistula. In these cases, massive intestinal bleeding may occur due to enteric erosion and bleeding or graft erosion with direct aortic bleeding inside the hollow viscera. 15 Although open repair with in situ reconstruction is the preferred choice for abdominal aortic graft infection, in cases of acute bleeding or aorto-enteric fistula, endovascular repair may be the most adequate first-line strategy. 16 17 This may be used as a bridge prior to definitive repair or as a permanent repair in cases with limited signs of infection and high surgical risk. If the latter option is chosen, after stabilization of the patient, bowel repair should be performed additionally. If used as bridging strategy, endovascular repair allows for hemodynamic stabilization, local and systematic infection treatment, and optimization of comorbidities, after which a definitive repair with in situ reconstruction and bowel repair may be performed. Antibiotic management in these cases is paramount and may vary according to the patient and hospital characteristics and the respective microbial flora. 17 18

## Endovascular Repair of Late Thoracic and Thoracoabdominal Aortic Open Repair Complications

### Chronic Type A Aortic Dissections Requiring Reintervention after Proximal Aortic Repair

Type A aortic dissection is a surgical emergency, and most patients receive open repair with ascending aortic or ascending aorta and arch replacements, with or without concomitant aortic valve repair or replacement. 19 These repairs allow for the management of the acute life-threating event by avoiding aortic rupture, cardiac tamponade, aortic insufficiency, supra-aortic vessel compromise, and coronary artery involvement. 19 Additionally, they may completely resolve the aortic dissection if this is limited to a short aortic segment. However, frequently an area of residual dissection is usually left, which may then in the future degenerate or complicate and thus require further reinterventions. 20 21 22 23

Open repair in these cases is associated with high degrees of morbidity and mortality. Gaudino et al 24 have shown in a systematic review and meta-analysis that aortic reoperation occurs at a mean of 2.4% per person-year in the 5 years following initial ascending aortic repair. The main reason for aortic reoperation was aortic dissection as the initial diagnosis. In the meta-analysis, the pooled in-hospital mortality was 14% and complication rate was 18% following open reoperation. Specific complications such as neurologic events occurred in 14.7%, acute renal injury requiring dialysis in 8.8%, and prolonged mechanical ventilation in 44.1%.

In these cases, endovascular repair with an endovascular arch device may be a good option if adequate length of the ascending aortic graft is left to deploy the graft. Endovascular repair has the benefit of being less invasive and avoiding the need for a redo sternotomy. However, endovascular repair of the proximal aorta is complex and should be performed only in experienced centers.

In a recent multicenter study by Verscheure et al, 20 using the COOK a-branch (COOK Medical, Bloomington, IN) for treatment of chronic post-Type A aortic dissections following proximal aortic repair, technical success was 94.3%, with perioperative mortality of 2.9% and permanent stroke rate of 2.9%. These are excellent results when compared with open surgery. Furthermore, studies have shown that approximately 70% of patients with chronic Type A aortic dissections with previous proximal repair are feasible for endovascular aortic arch repair. 25

**Fig. 2:**
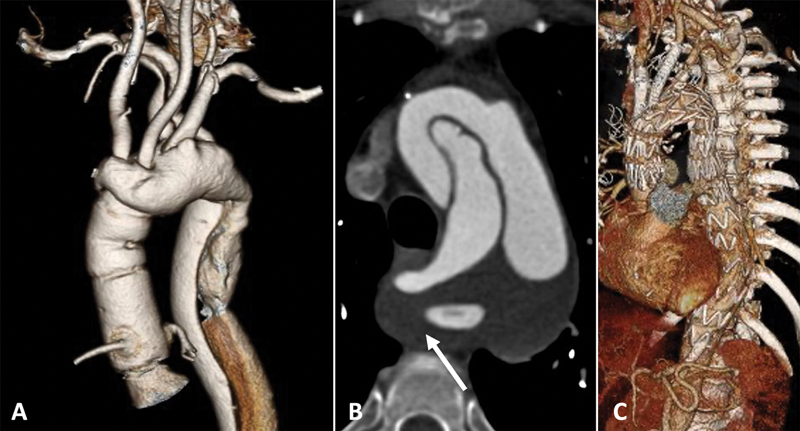
Patient with a chronic residual aortic arch dissection with aneurysmal degeneration following proximal aortic repair of a Type A aortic dissection in a patient with right aberrant subclavian artery and Kommerell's diverticulum. ( **A** ) Three-dimensional (3D) reconstruction of the preoperative computed tomography (CT) angiography demonstrating the aortic arch postdissection aneurysm and Kommerell's diverticulum. ( **B** ) Axial view of the CT angiography showing the aortic arch postdissection aneurysm and Kommerell's diverticulum ( *arrow* ). ( **C** ) 3D reconstruction of the postoperative CT angiography after endovascular aortic arch repair. A COOK a-branch with two inner branches for the right common carotid artery (CCA) and left CCA with additional left CCA to left subclavian bypass and right CCA to right subclavian bypass, with proximal occlusion of both subclavian arteries using plugs.

In Fig. 2 , we illustrate a case in which an endovascular repair was used to treat a post-type A chronic aortic arch dissection using a COOK a-branch with two inner branches for the common carotid arteries. The patient had been previously submitted to a proximal aortic arch repair and developed an aneurysm in the residual dissection at the arch and descending thoracic aorta. Additionally, the patient also had a right aberrant right subclavian artery with a Kommerell's diverticulum; therefore, a right common carotid artery (CCA) to right subclavian artery and left CCA to left subclavian artery bypass were performed previously, followed by occlusion of both subclavian artery origins using endovascular plugs.

A possible significant limitation in these patients may be the existence of a mechanical aortic valve. In a feasibility study analyzing the use of endovascular arch repair following Type A aortic dissection, short and kinked proximal grafts in addition to aortic mechanical valve were found to be a significant limitation for endovascular arch repair, arguing for a higher awareness of these issues in the index repair. 26 Although mechanical aortic valve may increase the complexity of the repair, we have shown this may be overcome by using a custom-made short tip delivery sheath and by advancing the tip on periphery of the valve, passing only through one cusp and thus avoiding advancing through the middle, which may cause severe aortic insufficiency and damage the aortic cusps. 27 This is in contrast to biological valves that, although require a more delicate manipulation than normal valves, do not present an issue for crossing devices.

**Fig. 3:**
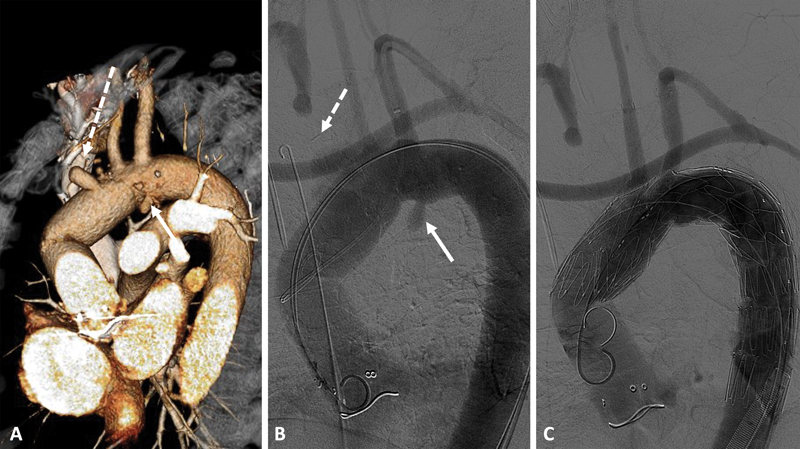
Patient with a suture line pseudoaneurysm ( *arrow* ) after previous proximal aorta and hemiarch repair, with chronic occlusion of the brachiocephalic trunk ( *dotted arrow* ) submitted to an endovascular arch repair using a COOK a-branch with two inner branches for the left common carotid artery and left subclavian artery with additional left axillary artery to right axillary artery bypass (performed in a staged repair) to revascularize both the right cerebral hemisphere and upper limb. ( **A** ) Preoperative three-dimensional computed tomography angiography reconstruction. ( **B** ) Initial aortic arch angiogram showing the suture line pseudoaneurysm ( *arrow* ) and the occlusion of the brachiocephalic trunk ( *dotted arrow* ). ( **C** ) Final angiographic control of the endovascular aortic arch repair (note that the proximal sealing was achieved before the sharp angulation of the previous anastomosis between the hemiarch repair and the proximal ascending aortic repair).

Para-anastomotic pseudoaneurysms may also occur after open aortic arch repairs. In these cases, endovascular repair has the advantage of avoiding a resternotomy and the morbidity associated with open aortic arch reintervention. In Fig. 3 we illustrate a case in which a false aneurysm developed on the suture line of a previous hemiarch repair with concomitant occlusion of the innominate artery branch. This was resolved by performing a left axillary to right axillary bypass followed by endovascular repair with a COOK a-branch with two inner branches for the left carotid and left subclavian artery (note that the proximal sealing was achieved before the sharp angulation of the previous anastomosis between the hemiarch repair and the proximal ascending aortic repair).

### Reinterventions after Open Thoracoabdominal Aortic Repair

Need for reinterventions after open thoracoabdominal (TAAA) and descending thoracic aortic repairs is not infrequent. 28 Latz et al 29 have analyzed the outcomes of 516 open repairs of extent I-III TAAAs. At a mean follow-up of 4.4 years, 98 patients (19%) developed late aortic and graft-related events.

In these cases, open reinterventions have been shown to be associated with very high rates of morbidity and mortality. 28 30 Even in very high-volume and experienced centers, early mortality is as high as 23%. 28

During open TAAA repair it is frequent to include the visceral aortic vessels as an island patch, frequently called the Carrel patch. 30 Also, intercostal arteries may additionally be revascularized during TAAA repair with an intercostal patch reimplantation. 30 During follow-up, however, disease progression in this remaining native aortic tissue may also occur, necessitating reintervention. Dardik et al 31 found that during follow-up of 107 patients submitted to open TAAA repair with visceral vessel incorporation using the Carrell patch, patch aneurysmal expansion occurred in 7.5% ( *n* = 8). When analyzing only patients with connective tissue disease, this occurred in 18%. Of these eight patients, five were submitted to open repair, of whom two died intraoperatively. Patch aneurysmal expansion has also been reported to occur in intercostal artery patch revascularizations. 32

Bertoglio et al 33 have analyzed the different results obtained from open, hybrid, and endovascular repair of visceral aortic patch aneurysms after TAAA repair. In this study they found that endovascular repair was associated with lower adverse events and mortality than both open and hybrid surgeries, favoring this option if feasible.

Recently, the Trans-Atlantic Aortic Consortium has published a multicenter study analyzing the outcomes of endovascular repair of intercostal and visceral patch aneurysms following open TAAA aneurysm repair. Overall, 29 patients were included, 24 patients treated using custom-made fenestrated and/or branched devices, 3 patients with the off-the-shelf T-branch (COOK Medical devices), and 2 patients with physician-modified endografts. A total of 103 target vessels were included, 54 with fenestrations, and 49 with directional branches. Technical success was achieved in 100% with no 30-day or in-hospital mortality. Primary and secondary patency rates at 2 years for target vessels were 95% and 100%, and freedom from target vessel instability was 83%. However, freedom from reintervention at 2 years was only 61% (albeit most being endovascular percutaneous procedures under local anesthesia) highlighting the need for regular surveillance in these patients. 34

**Fig. 4:**
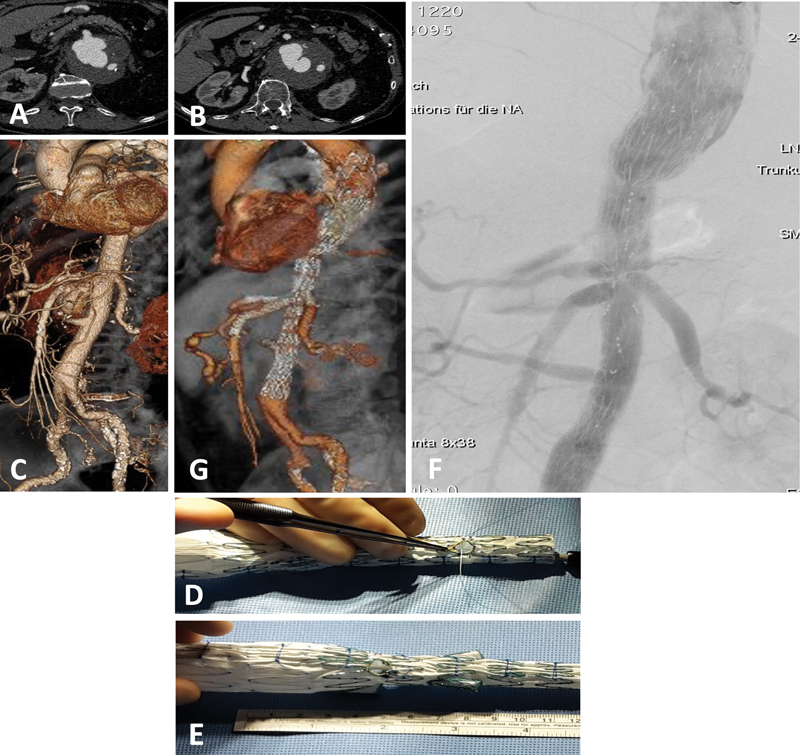
Patient with a prior open thoracoabdominal aortic repair who developed a visceral patch aneurysmal degeneration with additional para-anastomotic aneurysm at this level. The patient had left renal bypass at the level of the visceral patch and the right renal had been reimplanted 4 cm below the visceral patch. To address the anatomic constrains a physician modified T-Branch (COOK) was used by creating two additional fenestrations, one on the opposite side at the level between the celiac and mesenteric branch (intended for the left renal artery) and the other 4 cm below the visceral branches (intended for the right renal artery). At the end of the procedure both original renal branches were occluded with endovascular plugs. ( **A** and **B** ) Preoperative computed tomography angiography (CTA) demonstrating the visceral patch para-anastomotic aneurysm. ( **C** ) Three-dimensional (3D) reconstruction of preoperative CTA. ( **D** and **E** ) On table modification of the T-branch device with fenestration reenforcement using a double-looped goose-neck snare sutured with 4–0 ethibond suture. ( **F** ) Final angiographic control. ( **G** ) Postoperative 3D CTA reconstruction.

In Fig. 4 , we illustrate a case of a 64-year-old patient with prior open TAAA repair who developed a patch aneurysmal degeneration with additional para-anastomotic aneurysm at this level. This patient was treated with a physician-modified graft by modifying a COOK T-Branch with two additional fenestrations for the renal arteries and occluding both renal branches.

Prevention of spinal cord ischemia in these patients is highly relevant. If patients had intercostal artery preserving techniques, one should always protect this revascularization whenever possible by avoiding extensive proximal coverage. If this is not possible, testing spinal cord perfusion may be performed by inflating a balloon at this level with neuromonitoring, as described by Trans-Atlantic Aortic Research Consortium Investigators, 34 to aid in decision making.

As described above, visceral patch aneurysmal degeneration is much more frequent in patients with connective tissue disease. This is challenging since endovascular repair has been classically avoided in these patients due to durability issues. However, since endovascular repair in these cases is performed with proximal and distal sealing in a previous synthetic graft (“graft-to-graft”), this approach may be a solution even for connective tissue disease patients. In the Trans-Atlantic Aortic Consortium multicenter study, seven patients presented with connective tissue disease.

In patients with connective tissue disease, the main preoccupation with endovascular aortic repair is the high risk of continuous proximal or distal aortic dilation with loss of sealing zone and possible graft migration. 35 In cases already submitted to open repair, allowing for proximal or proximal and distal landing zones in synthetic grafts, endovascular repair may be a good option by overcoming this limitation. 35 36 Clough et al 35 showed that this strategy was both feasible and safe, although more mid- and long-term follow-up is needed to correctly assess durability of these repairs. Connective tissue disease patients are frequently burdened with multiple reinterventions, which significantly impairs their quality of life via anxiety related to surgeries, which may lead them to seek less invasive options.

### Thoracic Aortic Graft Erosion with Esophageal or Bronchial/Pulmonary Fistula

Following open repair of thoracic or thoracoabdominal aortic grafts, the continuous friction of the graft may lead to the development of graft erosion and fistulation to the esophagus (esophageal fistula) or bronchus/lung tissue (airway fistula). These patients may present with massive bleeding due to graft disruption or episodical bleeding due to bleeding from the esophagus wall or lung tissue directly, presenting as hematemesis or hemoptysis, respectively. In these cases, endovascular repair with a TEVAR may be lifesaving in the case of massive bleeding and may serve as a bridging therapy or as a definite treatment, depending on the patient overall status, tissues involved, and presence of graft infection. 15 37 38 39

In the case of esophageal fistula, the graft is almost always infected, and so, TEVAR may only be consider the definitive treatment option in patients who do not tolerate an open repair. In frail patients, a palliative option with TEVAR followed by lifelong antibiotic may be the best option. In most cases, however, a bridge repair (after TEVAR) with complete graft removal, esophagectomy, and in situ repair or extra-anatomical aortic repair is the best option. 15 38

In the case of an airway fistula, the graft may not be infected and following endovascular repair for the graft disruption the airway injury may be closed with endobronchial suture or exclusion or open pulmonary resection with graft preservation. 15 40 41

## Conclusion

Late aortic or graft-related complications after open aortic repair are not infrequent. Endovascular techniques can offer several possible solutions for late complications following open aortic repair. These may be tailored for the specific complication and usually are less invasive, with lower morbidity and mortality than open reinterventions. However, routine follow-up after these reinterventions is still needed as further procedures may be necessary.
